# Design of a Data Glove for Assessment of Hand Performance Using Supervised Machine Learning

**DOI:** 10.3390/s21216948

**Published:** 2021-10-20

**Authors:** Hussein Sarwat, Hassan Sarwat, Shady A. Maged, Tamer H. Emara, Ahmed M. Elbokl, Mohammed Ibrahim Awad

**Affiliations:** 1 Mechatronics Engineering Department, Faculty of Engineering, Ain Shams University, Cairo 11517, Egypt; Shady.Maged@eng.asu.edu.eg; 2 Faculty of Computer Science, Ain Shams University, Cairo 11566, Egypt; hassan.mahmoud@cis.asu.edu.eg; 3 Faculty of Medicine, Ain Shams University, Cairo 11591, Egypt; tamer_emara@med.asu.edu.eg (T.H.E.); ahmed.elbokl@med.asu.edu.eg (A.M.E.)

**Keywords:** data glove, home rehabilitation, machine learning, IoT

## Abstract

The large number of poststroke recovery patients poses a burden on rehabilitation centers, hospitals, and physiotherapists. The advent of rehabilitation robotics and automated assessment systems can ease this burden by assisting in the rehabilitation of patients with a high level of recovery. This assistance will enable medical professionals to either better provide for patients with severe injuries or treat more patients. It also translates into financial assistance as well in the long run. This paper demonstrated an automated assessment system for in-home rehabilitation utilizing a data glove, a mobile application, and machine learning algorithms. The system can be used by poststroke patients with a high level of recovery to assess their performance. Furthermore, this assessment can be sent to a medical professional for supervision. Additionally, a comparison between two machine learning classifiers was performed on their assessment of physical exercises. The proposed system has an accuracy of 85% (±5.1%) with careful feature and classifier selection.

## 1. Introduction

Stroke is the second cause of death worldwide and is the third cause of disability [1,2]. It happens when the blood supply to some portion of the brain is hindered or decreased, forestalling brain tissue from obtaining oxygen and nutrients, and within a few minutes, brain cells start to die. Prompt action needs to be taken to prevent injuries or death [3].

Poststroke injuries can be a brief or permanent handicap. This incorporates loss of muscle movement, trouble talking or gulping, emotional issues, cognitive decline, and/or memory loss. These injuries incredibly influence the everyday lives of stroke survivors, and while some recuperate the majority of their capacity, others need perpetual consideration [3]. Furthermore, the loss of ability can lead to poststroke depression, which influences roughly one-third of stroke survivors [4].

Stroke rehabilitation starts when the patient’s medical condition is stabilized and there is no danger of another stroke in the near future. Contingent upon the stroke severity, a few patients make a fast recuperation; others need months or years after their stroke. The cycle for the most part starts in the hospital and should then be possible in either a rehabilitation unit, skilled nursing facility, or even the patient’s home. It includes physical and cognitive exercises, which need the support of doctors, physical therapists, rehabilitation nurses, and more relying on the patient’s condition. It is a recurrent cycle that includes: assessment, goal setting, intervention, and lastly, reassessment [5].

Numerous aspects of the patient’s physical and mental performance need recurring assessment over the rehabilitation period. Common assessment techniques include the Fugl–Meyer [6] for the evaluation of motor functions, the Boston Diagnostic Aphasia Examination [7] for the assessment of speech and language, and many others for the assessment of a wide array of functions. One of the common disadvantages among these methods is the requirement for a physician to administer the test. The spread of COVID-19 has highlighted the need for automated in-home rehabilitation and assessment systems [8].

Automated assessment systems can be utilized to reduce the number of visits to a physician for assessment. These systems either perform an analysis of the data [9] or use a machine learning classifier [10]. The use of machine learning in patient diagnosis and assessment has been on the rise [11]. However, it cannot replace the diagnosis of a trained physician [12] and is employed when a physician is not available. The employed algorithms need to demonstrate reliable results when diagnosing and handling absent and noisy data. Additionally, the yielded results should be explicit to the physician. Many advanced machine learning models are thought of as “black boxes” since their complexity obscures them in human thinking. Therefore, physicians are reluctant to recognize them as it is difficult to relate the models to support hypotheses. Before testing them on patients, these models have to be approved by clinical experts [13].

Rehabilitation from a stroke injury is a financially and physically burdensome endeavor, which not everyone might be able to handle, without the needed amount of support. Hence, the advent of rehabilitation robotics is needed, as it increases the number of patients that physiotherapists can treat at a time, with usually a lower cost [14]. Additionally, the Internet of Things (IoT) can then be combined with rehabilitation robotics to monitor patients from their homes, to have their families motivate them [15].

These rehabilitation robotics and IoT systems will need an automated assessment system that can track the patients’ progress and encourage them to perform better. One example of an automated assessment system was included in [16], where a machine learning classifier, AdaBoost [17], was combined with wearable sensors to create an IoT-based upper limb rehabilitation assessment. Four commonly used joint actions generally performed in clinical assessments were performed and given a classification depending on the subject’s range of motion. Using the proposed algorithm, the average accuracy of the classifier was higher than 98%.

Another example of an automated assessment system for upper limb rehabilitation was presented in [18], which utilizes motion capture data using the Microsoft Kinect VS [19]. The motion of 25 joints of the patient is tracked and then placed into a feedforward-neural-network (FFNN)-based assessment model to calculate clinical scores. The proposed model had an overall accuracy ranging from 0.87–0.96 depending on the performed task. Other examples of automated systems were presented in [20,21,22].

The need for automated assessment and rehabilitation has been highlighted in recent studies [23,24,25]. Automated assessment systems offer a multitude of benefits to patient and physician alike, and they are expected to become a complementary tool for clinical use. Hence, the use of traditional assessment methods was encouraged for automated assessment systems, as they are still considered the “gold standard” for verifying the effectiveness of a treatment.

**Figure 1:**
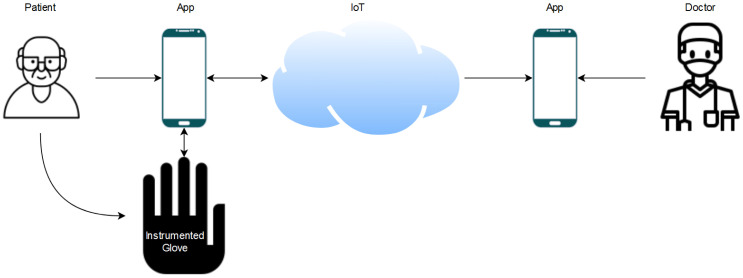
System overview.

This paper demonstrates the design of a data glove with a mobile application, a real-time database for uploading and retrieval of patient data, and a web server for assessing patient performance. The patient data were gathered using the data glove, which was outfitted with flex sensors, force-resistive sensors (FSRs), and IMUs. This is common in rehabilitation robotics as they can gather many kinematic data and can be paired with other frameworks. Furthermore, the performance of two machine learning algorithms was evaluated for in-home rehabilitation. A summary of the overall process is displayed in Figure 1. The goal was to determine the applicability of the data glove and machine learning in-home rehabilitation assessment. All algorithms utilized the same set of features extracted by the data glove. This is a continuation of our previous work on home rehabilitation and assessment [26,27].

The outline of this paper is as follows. The next section demonstrates the glove design and system integration, respectively. Afterward, a brief theoretical description of the employed machine learning algorithms is given before describing the utilized experimental protocols. Then, the results are showcased in Section 3 and discussed in the last section.

## 2. Materials and Methods

### 2.1. System

The system comprises the data glove for gathering the patient’s data, a mobile app for reviewing previous data and for performing new assessments to monitor the patient’s progress, a web server for computing the assessment of the patient and sending back the patient’s score to the app, and an online database to store the patient’s records.

The process begins with the patient starting the exercises through the mobile application. The application sends the exercise information to the Arduino microcontroller, via Bluetooth, and tells the patient to start the exercise. Based on the received information, the microcontroller reads and processes the data from the selected sensors.

**Figure 2:**
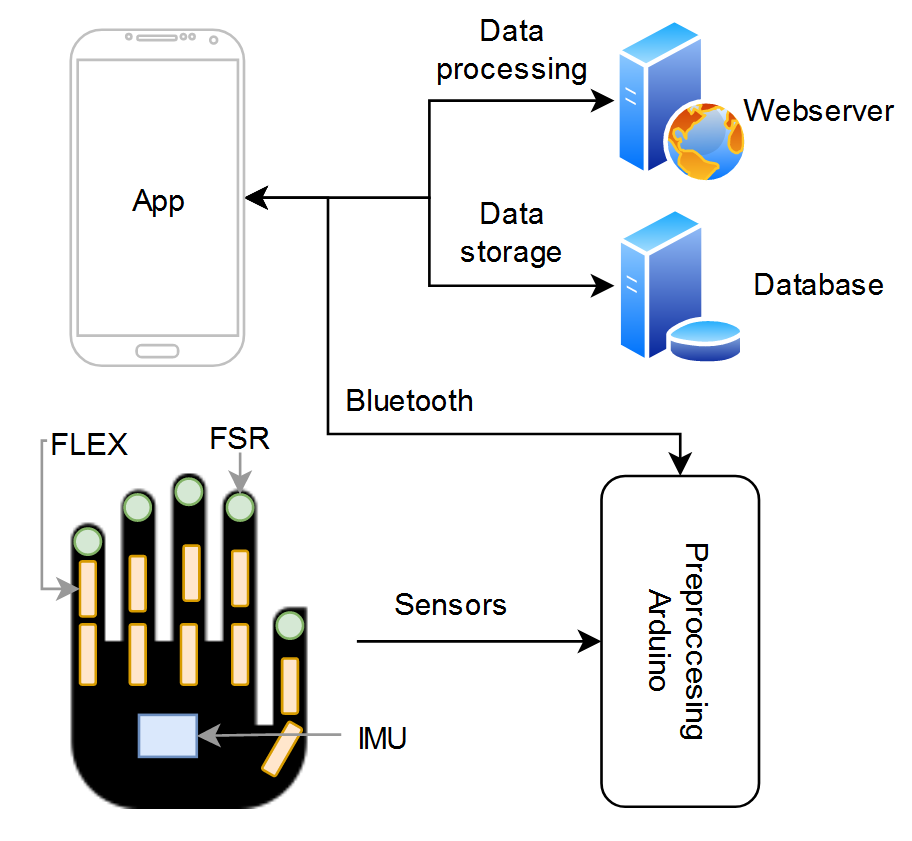
Integrated system.

After processing, the data are sent back to the application. The application uploads these data to the web server, where they are further processed and evaluated using machine learning algorithms. The output is then sent back to the application, where it can be uploaded and stored on the database for later viewing. The information on the database can also be accessed by a medical professional through the application to monitor the patient’s performance. The overall process is demonstrated in Figure 2.

### 2.2. Data Glove

For the data glove, we placed 10 flex sensors to determine the angles of each finger joint, 5 force-resistive sensors (FSR) for gripping force, and a 6-axis IMU to find the movement of the wrist. The placement of the sensors, displayed in Figure 2, was configured according to similar literature on hand gesture recognition [28,29,30,31]. The accuracy of similar data gloves with machine learning algorithms has been demonstrated in several papers [28,29]. The biggest downside, however, is the inaccuracy resulting from different hand sizes.

**Figure 3:**
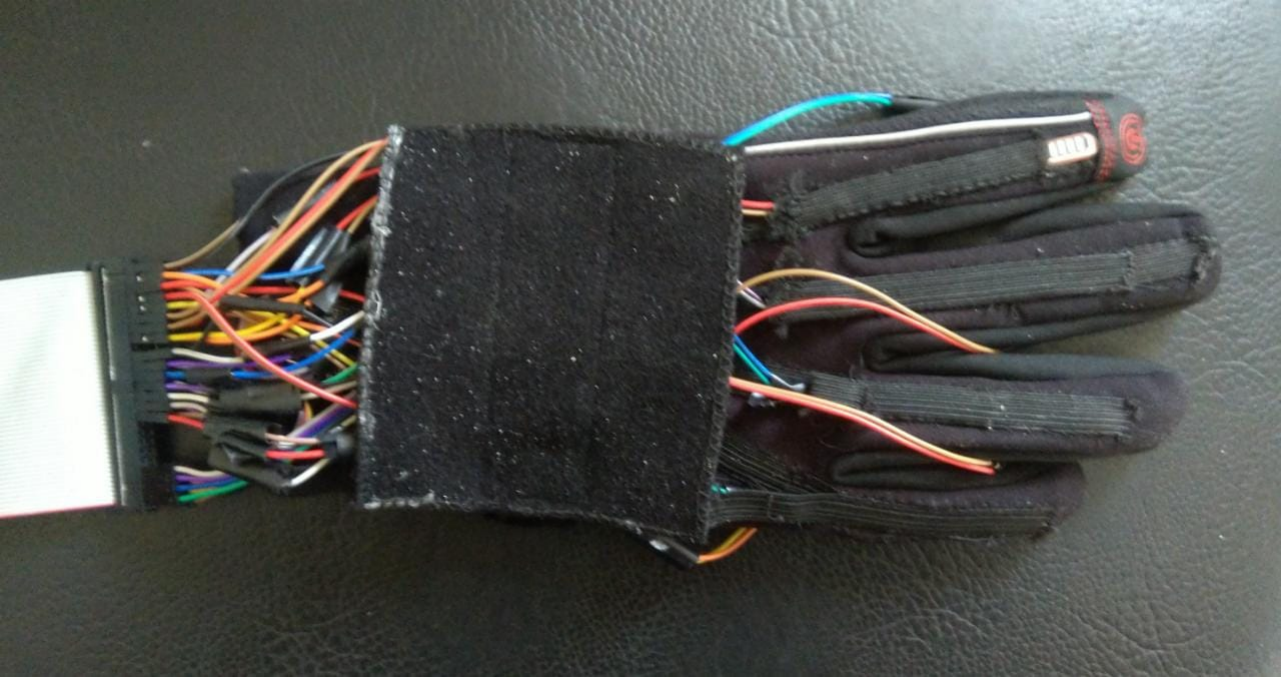
Data glove.

An image of the data glove is displayed in Figure 3, where the sensors are knit into the glove to provide a more aesthetic appearance. Two flex sensors and one FSR are placed on each finger. The flex sensors measure the bending angle of the metacarpophalangeal joints and proximal interphalangeal joints, while the FSRs are placed on the fingertips. Flex sensor *F1* is placed on the metacarpophalangeal joint for the thumb, whilst *F2* is placed on the interphalangeal joint. The same pattern follows for the rest of the fingers.

### 2.3. Sensors

The flex sensor is used to determine the angle of each finger joint [32]. The sensor works as a voltage divider with a flat resistance at the normal bend; increasing the bend increases the resistance. With some calibration, this value can be used to determine the angles of each finger joint. Unfortunately, this sensor fails to tell the overall orientation of the hand and wrist, which is why an IMU sensor was also employed.

**Figure 4:**
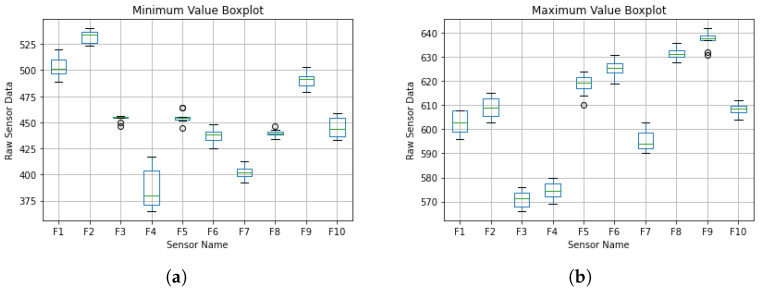
Box and whisker plot of sensor readings for the calibration of the bending. (**a**) Minimum Value, occurs during maximum bend, (**b**) Maximum Value, occurs during initial Pose.

To test the repeatability of the flex sensor, the raw data at the starting position and at maximum deflection of the fingers were recorded for 10 trials. A box and whisker plot of the sensor readings is displayed in Figure 4.

To measure the gripping force, an FSR was placed on the distal phalanx for each finger [33]. The FSR was less accurate due to the small range of forces utilized. Therefore, the maximum reading that the patient reaches can give insight into his/her gripping force, with the average person providing readings for 700 and higher. No calibration was required for this sensor, and only the raw data were taken for feature extraction.

The IMU sensor (MPU6050) uses a tri-axial accelerometer and a tri-axial gyroscope to determine the orientation [34]. The IMU sensor was placed on the back of the hand to determine the orientation of the wrist, which is crucial for determining the wrist’s current pose. Median filtering was implemented to smoothen the data from the IMU [35].

### 2.4. Microcontroller

An Arduino Mega was used as the microcontroller due to the abundant inputs and outputs needed to operate the glove and transmit the data. Additionally, since the machine learning and classification were performed using an external web server, the computing power of the Arduino was sufficient to handle all the required tasks. These tasks included reading data from the sensor, calibrating the data to reduce error, feature extraction, and receiving and transmitting data to the mobile app using Bluetooth serial communication.

### 2.5. Feature Extraction

The readings from each sensor are displayed in Table 1.

For the flex sensor, the mean, the root-mean-squared (RMS), the standard deviation, and the minimum value were used. For the FSR, the mean, the RMS, the standard deviation, and the maximum value were taken. Finally, features usually utilized in similar methodology and procedures were extracted from the IMU, due to their statistical representation of large datasets [36]. These features included: the mean, the RMS, and the standard deviation for each axis of the accelerometer and gyroscope, as well as the signal magnitude area (SMA) of the accelerometer and gyroscope.

The equation used to calculate the SMA for the accelerometer and gyroscope was:

(1) $$f_{\mathrm{SMA}}=\frac{1}{T_{total}}\int_{0}^{T_{total}}\left|x\left(t\right)\right|+\left|y\left(t\right)\right|+|z\left(t\right)|dt$$

The SMA is the standardized integral of the original values, given by partitioning the region under the curve with the time interval of the readings [37].

### 2.6. Mobile Application

The mobile application, called iGrasp, was developed using MIT App inventor, a web-based block coding development tool for Android phones [38]. This open-source development tool was developed by Google and is currently maintained by MIT.

The purpose of the application was to monitor the patient’s progress and check for any abnormalities, which is why the following features were added:

- Patients can view their score of previous attempts;
- Patients can connect to the data glove and upload the scores of new attempts;
- Doctors can view their patients’ uploaded scores.

When starting the application, the user first registers as either a patient or a doctor. Registering as a doctor will permit him/her to retrieve patient data from the database, whilst registering as a patient will make it possible for him/her to assess his/her performance. The user can store his/her performance score for later viewing by uploading it to the database, or if he/she finds the outcome unsatisfactory, he/she can retry his/her attempt as many times as needed. While performing the exercises, the application displays helpful images and instructions to guide the user on how to perform the exercise.

**Figure 5:**
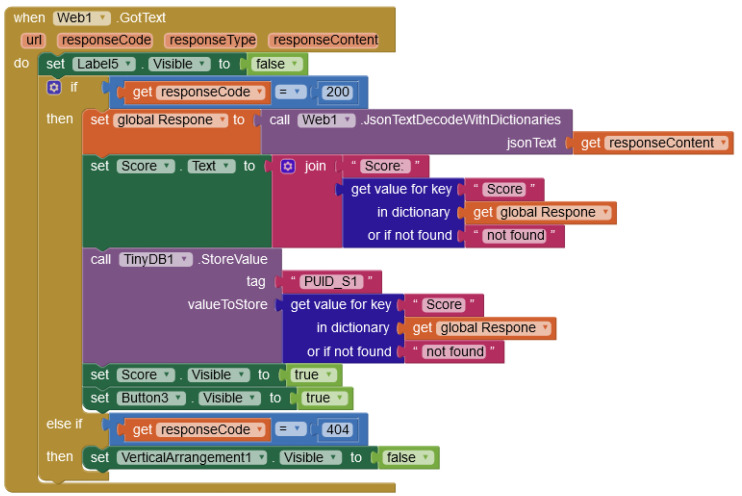
In this block, the application reads the web response and stores it in the phone’s memory, where it can be uploaded later. The simplicity of block-based coding makes it available for use and editing without the need for a skilled technician.

The mobile application connects with the data glove via Bluetooth. The communication protocol starts with the mobile application sending the exercise that the subject is currently performing. The microcontroller then records the data from the needed sensors, extracts the features, and then sends the extracted features to the app as a JSON text file. The application sends the JSON file to the web server for assessment before retrieving the score and displaying it to the patient. An example block is displayed in Figure 5.

### 2.7. Web Server and Database

The web server was written using Flask API [39]. It is a scalable lightweight web server Gateway Interface (WSGI) web application framework design for simple web applications. The function of our web application was to receive the patient’s data from the mobile application, which it received from the data glove, and run the employed machine learning model to assign a score for the patient. The web application will then output a JSON file that the mobile application reads and displays to the patient.

The web server was hosted using Heruko Platform [40]. This platform was employed for the following reasons:

- It offers Python support;
- It supports the employed machine learning model;
- It has simple steps to run and deploy the web application;
- It is reliable and does not require constant maintenance or updating;
- It is scalable and cost-efficient.

**Figure 6:**
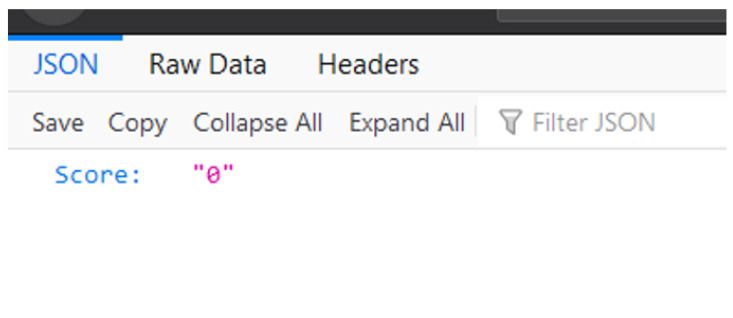
Output JSON file.

The web server did not have a direct user interface and was only meant for interaction with the mobile application. Other attempts at visiting the website would lead to a 404 error. The web application would only output a JSON file as the one displayed in Figure 6. This JSON file displays the patient’s score for the attempted exercise.

### 2.8. Database

For the database, we used Firebase [41]. Developed by Google, it is a NoSQL database that synchronizes data in real-time to all connected devices on a cloud. This platform is designed for mobile and web applications and was utilized in this work due to its real-time database feature, allowing us to update and store patients’ records worldwide.

Each user can preview the history of his/her attempts, including to monitor his/her progress. The database also makes it possible for physiotherapists and doctors to monitor their patients, if they have access to the patients’ usernames on the app.

### 2.9. Machine Learning

#### 2.9.1. XGBoost

EXtreme Gradient Boosting (XGBoost), an open-source boosting system, was one of the algorithms utilized to assess the patient [42]. Gradient boosting, in general, is a gathering strategy where every tree boosts the elements that prompted the miscategorization of the previous tree, by giving a continuous score for each leaf $w_{i}$. For a given example, the decision rules are used to categorize the leaves and calculate the final prediction by adding up the score in the corresponding leaves (given by $w_{i}$).

The regularized objective can then be:

(2) $$L\left(\phi \right)=\sum_{i}l\left({\hat{y}}_{i},y_{i}\right)+\sum_{K}\Omega \left(f_{k}\right)$$

where:

(3) $$\Omega \left(f_{k}\right)=\gamma T+\frac{1}{2}\lambda {∥w∥}^{2}$$

l represents a differentiable convex loss function that evaluates the distance between the expected value ${\hat{y}}_{i}$ and the goal value $y_{i}$. $\Omega (f_{k})$ denotes a regularization term that helps in preventing overfitting, where $f_{k}$ and *T* denote an independent tree system and the number of leaves in a system, respectively. A similar regularization technique was used in [43].

The model was trained in an additive manner, due to the inability of traditional methods to optimize the tree ensemble model in Equation (2), since it includes functions as parameters. Let ${\hat{y}}_{i}^{\left(t\right)}$ denote the prediction of the *i*-th instance at the *t*-th iteration. $f_{t}$ must be added to minimize the objective:

(4) $$L^{\left(t\right)}=\sum_{i=1}^{n}l\left(y_{1},{\hat{y}}_{i}^{\left(t-1\right)}+f_{t}\left(x_{i}\right)\right)+\sum_{K}\Omega \left(f_{t}\right)$$

Second-order Taylor expansion was employed to optimize the function. The constant parts were then removed, and the minimized objective at *t* is:

(5) $${\tilde{L}}^{\left(t\right)}=\sum_{i=1}^{n}\left[g_{i}f_{t}\left(x_{i}\right)+\frac{1}{2}h_{i}f_{t}^{2}\left(x_{i}\right)\right]+\Omega \left(f_{t}\right)$$

where $g_{i}$ and $h_{i}$ denote the first- and second-order gradient statistics on the loss function, respectively. The corresponding optimal value can be evaluated, after calculating the weight of leaf *j* for a fixed structure q(x), using:

(6) $${\tilde{L}}^{\left(t\right)}=-\frac{1}{2}\sum_{j=1}^{T}\frac{{\left(\sum_{i\in I_{j}}g_{i}\right)}^{2}}{\sum_{i\in I_{j}}h_{i}+\lambda }+\gamma T$$

Equation (6) can be used to assess the performance of a tree structure q.

XGBoost uses a modified version of the the greedy algorithm, called the approximate greedy algorithm [44]. This version is suitable for large sets of data as it decreases the number of thresholds that need to be observed, choosing instead to separate the data into quantiles based on feature distribution. This is improved even further by the use of a weighted quantile sketch. The sketch bases the difference between each quantile on weight, not observation, where the weight of each observation is $h_{i}$, meaning the quantiles are partitioned where the sum of the weights is similar. Therefore, quantiles are generated in areas with low confidence.

XGBoost uses a sparsity-aware-split-finding algorithm to handle missing data. This works by adding a default direction in each tree node and having the missing values classified. The default direction is chosen based on previous data. Moreover, the algorithm is able to treat new observations with missing values.

#### 2.9.2. Logistic Regression

Logistic regression [45] seeks to classify an observation by estimating the probability that an observation is in a particular category based on the values of the independent variables, which can be continuous or not.

The dependent variable in logistic regression follows the Bernoulli distribution having an unknown probability *p* where success is classified as 1 and failure as 0, so the probability of success is *p* and failure is q=1−p [46]. In logistic regression, we estimate *p* for any given linear combinations for the independent variables.

To tie together our linear combination of variables, we needed a function that links them together and essentially maps the linear combination of variables that could result in any variable onto the Bernoulli probability distribution where p∈(0,1).

The natural logarithm of the odds ratio is the function:

(7) $$\ln odds=\ln\frac{p}{1-p}=logit\left(p\right)$$

However, in our logit function, 0 to 1 ran along the x-axis, but we wanted our probabilities on the y-axis; we can achieve this by taking the inverse of the logit function.

(8) $$logit^{-1}\left(\alpha \right)=\frac{1}{1+e^{-\alpha }}=\frac{e^{\alpha }}{1+e^{\alpha }}$$

where α equals the linear combination of our independent variables and their coefficients. The natural logarithm of the odds ratio is equivalent to a linear function of independent variables. The antilog of the logit function allows us to find the estimated regression equation:

(9) $$logit\left(p\right)=\ln\frac{p}{1-p}=\beta_{0}+\sum \left(\beta_{i}*x_{i}\right)$$

(10) $$antilog=\frac{p}{1-p}=\exp\left(\beta_{0}+\sum \left(\beta_{i}*x_{i}\right)\right)$$

(11) $$\hat{p}=\frac{\exp(\beta_{0}+\sum \left(\beta_{i}*x_{i}\right))}{1+\exp(\beta_{0}+\sum \left(\beta_{i}*x_{i}\right))}$$

where $\beta_{0}$ and $\beta_{1}$ are the coefficients for the bias and weights, respectively. In order to update the coefficients $\beta_{i}$, we used the maximum likelihood:

(12) $$\mathrm{likelihood}=\hat{p}*p+\left(1-\hat{p}\right)*\left(1-p\right)$$

and then transformed the likelihood function using the log transform:

(13) $$\log\left(\mathrm{likelihood}\right)=\log\left(\hat{p}\right)*p+\log\left(1–\hat{p}\right)*\left(1-p\right)$$

Finally, we can sum the likelihood function across all examples in the dataset to maximize the likelihood:

(14) $$F_{max}=\sum_{i=1}^{n}(\log\left(\hat{p_{i}}\right)*p_{i}+log\left(1–\hat{p_{i}}\right)*\left(1–p_{i}\right)$$

It is common practice to minimize a cost function for optimization problems; therefore, we can invert the function so that we minimize the negative log-likelihood:

(15) $$F_{min}=-\sum_{i=1}^{n}\left(log\left(\hat{p_{i}}\right)*p_{i}+\log\left(1–\hat{p_{i}}\right)*\left(1–p_{i}\right)\right)$$

Calculating the negative of the log-likelihood function for the Bernoulli distribution is equivalent to calculating the cross-entropy function for the Bernoulli distribution, where $c_{i}$ represents the probability of class i and *d* represents the estimation of the probability distribution, in this case by our logistic regression model.

(16) $$F_{crossentropy}=-\sum \left(\log\left(d\right)*c_{i}\right)$$

### 2.10. Experimental Protocols

The study was conducted in accordance with the Declaration of Helsinki, and the protocol was approved by the University Ethical Review Board. All subjects gave their informed consent for inclusion before they participated in the study.

To assess the performance, the participants were asked to perform three tasks, recommended by a rehabilitation physician. These tasks were used to measure fine and gross patient performance, as well as any spasticity the patient may be suffering. This system was targeted towards subjects with a high level of recovery, who can continue their rehabilitation in-home.

All tasks started with the same initial hand pose, an open palm with the backside facing the participant’s face. Each task was repeated five times, not including practice trials. This number was suitable for assessment, as per [47]. Each trial lasted for five seconds, and the subjects were asked to complete each task and then return to the starting pose at a comfortable pace. Participants were scored from 0 to 2 for each exercise depending on their performance.

The first task, grasping, involved the subjects flexing all their fingers to the maximum, before returning to the initial pose. If the subject was unable to move, then the attempt was given a score of 0. If there was little movement, not all fingers were flexed, or the subject was unable to return to the resting position, then the attempt was scored as 1. If the subject achieved full motion and returned to the resting position, then the attempt was scored as 2. The flexion of the finger joints was the only thing measured during this exercise.

In the second task, pinching, the participant was asked to pinch his/her fingers and exert as much force as he/she could, then he/she should revert back to the initial position. No movement during the attempt resulted in a score of 0. Incomplete motion or inability to pinch (i.e., weak or no force exerted) or return to the initial position was scored as 1. Complete motion yielded a complete score of 2. The measurements for this exercise included the flexion of the thumb and index finger, as well as the pinching force.

**Figure 7:**
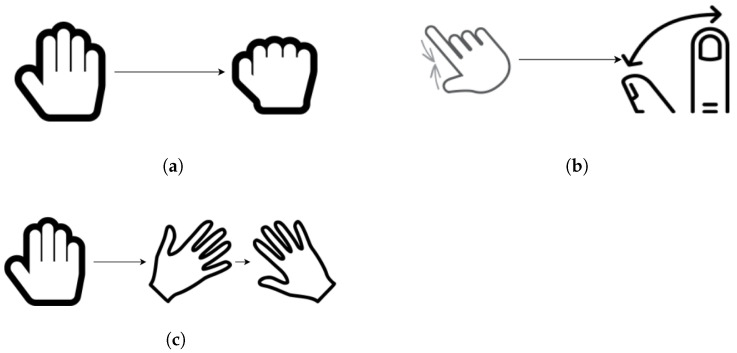
Exercise protocols. (**a**) Grasping, (**b**) Pinching, (**c**) Waving.

The final task involved waving. The participant was required to flex his/her wrist and then wave repeatedly for the duration of the exercise. The motion should be performed at a normal speed, and the participant did not revert back to the resting position. Each attempt was scored depending on the movement of the participant. Zero was awarded in the case of no movement and one in the case of little or some movement, incomplete flexion of the wrist, or complete flexion, but no waving. Finally, two was awarded if the subject completed the full motion for the duration. The orientation of the palm, the flexion of the wrist, and the acceleration were measured during this exercise. The tasks are displayed in Figure 7.

### 2.11. Patients

The patients used for this study, gathered from a rehabilitation center, all suffered from varying degrees of upper-extremity weakness. All three patients were able to perform the first exercise (grasping), but only Patients 1 and 3 were able to perform the second and third exercises. The patients’ information is displayed in Table 2. A supervising physician was available during the data gathering and provided the FMA score for the exercises performed.

## 3. Results

Two different machine learning classifiers, XGBoost and logistic regression, were employed and compared to test the most efficient algorithm. These classifiers were selected based on their visibility, which makes it easier for clinicians to understand their process, hence predicting where errors might occur. Each algorithm was repeated five times for each exercise to test possible feature combinations. The feature sets for each combination were selected randomly, with the first feature set including all the features. The dataset was gathered using four healthy right-handed subjects, three male and one female, with a mean age of 36.5±17.8 y. The healthy subjects were asked to perform the exercise with varying degrees of success and were scored from 0–2, as per the Fugl–Meyer assessment.

Both classifiers started with the same random state, and the test set was taken as 30% of the total dataset. The XGBoost classifier had a maximum tree depth of 3, and a 0.7 learning rate, whereas the logistic regression classifier used multinomial regression and had a maximum iteration of 100. The performance of each classifier was then tested using five-fold cross-validation [48].

For the first exercise, only the 10 flex sensors placed on the finger joints were utilized. These features extracted from the flex sensors were the minimum, mean, standard deviation, and root-mean-squared. Table 3 displays the k-fold accuracy for the combination of features for XGBoost (XGB) and Logistic Regression (LR).

The second exercise utilized the flex sensors and FSRs placed on the index and thumb fingers. The same features for the flex sensors were also extracted for the FSRs, except the minimum value was replaced with the maximum value of the FSR. The results are displayed in Table 4.

For the last exercise, waving, only the IMU placed on the backhand was utilized to record the data. The results are displayed in Table 5.

The produced models were then tested on the three patients, and the results were compared with the physician’s score. The performance of the XGB and LR models in comparison with the physician’s score are displayed in Table 6 and Table 7, respectively, with the best performing feature combination (BPF) outlined as well.

## 4. Discussion

To assess patient performance, the results outlined in the previous section demonstrated the need for the careful selection of a classifier. We can observe that for the healthy subject, the LR classifier outperformed the XGB classifier by a small margin. However, upon taking exercises performed by the patients, each classifier performed differently depending on the exercise. Since the selected exercises affect the selection of the classifier, the first option would be selecting the LR classifier due to its higher overall performance. Forfeiting the third exercise, or switching it with a similar one where the LR classifier performs better, would be the second option. Finally, the third option would be using both classifiers, despite the higher computational cost.

While utilizing all features often yields the highest accuracy, it is also possible to hyper-tune your features by testing their effect. For instance, in the waving exercise, the XGB classifier can achieve the same level of accuracy using only the STD and RMS as using all of them. Further testing on each exercise can lead to specific feature selection; this decreases the computational time and increases the sampling rate [49,50,51].

The accuracy of the classifiers expectedly decreased when tested on patients with upper-extremity injuries. This was due to the irregularity of the motion and the difficulty of replicating with healthy subjects for dataset training. Nevertheless, when optimizing the classifier and feature selection, the average system accuracy reached 85% (±5.1%). This is a clinically acceptable accuracy in comparison with other automated systems.

Table 8 presents a comparison between our system and other systems for automated upper-body assessment. The comparison involved the equipment used, the data gathered, and how patients were assessed and classified. Almost all employed machine learning algorithms for classification. Arguably, machine learning was not suitable for classification due to the lack of sizeable datasets. Therefore, the selection of scalable machine learning classifier was important. This highlights the benefits of classifiers such as XGBoost, which utilize transparent classification, which can be assessed by the physician. IoT monitoring checked if the results could be transmitted to a supervising physician, and the setting was where this system could be implemented. Finally, the accuracy was the average accuracy of all exercises tested on the patients.

The study in [18] developed an automated system that assesses patients using a feedforward neural network [54]. The Kinect V2 was employed due to its markerless 3D vision system, as well as its reliability in clinical settings [55]. The patients performed four tasks from the streamlined Wolf Motor Function Test (WMFT), the WFMT Functional Ability Scale (FAS). They were limited by their use of only the Kinect V2, but argued that the convenience was better and that the use of wearable sensors was not without limitations, as discussed above.

The automated assessment system in [21] utilized motion data from a cellphone’s IMU and visual data from the rear camera to reduce drift, leading to more accurate position information. The data were then wirelessly transmitted to a computer for feature extraction and automated assessment. The assessment was performed using a decision tree approach. The lack of calibrations, sensor placement, and low cost make this an effective tool. Nevertheless, it was unable to measure finger flexion/extension and gripping force.

The system proposed in [22] involved the use of several inexpensive pieces of equipment to streamline the FMA assessment procedure, reducing the total assessment time by 82%. Additionally, the equipment utilized can easily include most FMA assessment exercises. It is just a matter of adding new features and training a dataset. The dataset was trained using Support Vector Machine (SVM) [56]. Despite its low accuracy with individual tests, the total scores achieved were at least within 90% accuracy to that assigned by a physician. The accuracy on healthy subjects was much higher in comparison to stroke patients, especially for exercises that obtained a score of one, as the range of motion was hard to predict, whereas scores of zero and two, unable to perform and full completion, respectively, were much easier to predict due to the minor variations.

The data glove in [28] boasted a modular design that fixed some of the limitations of wearable sensors. This glove can fit onto patients with different hand sizes if care is taken in the placement of its 16 IMU sensors. It uses a quaternion algorithm to extract the hand’s acceleration, angular velocities, and joint angles. Then, it transmits the data via Bluetooth to a computer for assessment. It classifies patients using k-means clustering based on their Brunnstrom stage and is reasonably accurate at Stages 4 and 5 [57]. At Stage 6, most stroke patients perform similarly to healthy subjects, making it difficult to differentiate. Additionally, they visualized hand movements to help give insights to physicians on the patient’s movement.

Most upper-extremity automated assessment systems use the Kinect for data gathering and the FMA for assessment, with differing exercises, features, and/or classification methods [58,59,60,61]. This can be attributed to the ease of setting up the Kinect, while still automating most of the FMA with reliable accuracy. Furthermore, by adding a few more sensors, it is possible to fully automate the FMA. The disadvantage of such systems is the noise that could be produced in uncontrolled environments, reducing their reliability.

It can be observed that while there are many upper-limb automated assessment systems, few have developed in-home rehabilitation that has been clinically approved. The systems proposed in [16,62] were only tested on healthy subjects. IoT monitoring systems for elderly subjects usually monitor the patient’s movement for activity recognition [15,63].

The COVID-19 pandemic has highlighted the need for in-home rehabilitation systems, especially for care facilities for elderly people, where outbreaks can have a fatality rate of over 25% [64]. The work in this paper contributes an in-home automated assessment system for rehabilitation of upper-extremity patients with high recovery. The system utilizes IoT to allow for patient progression monitoring. This inexpensive system can be easily set up at home and only uses the demonstrated data glove and a mobile app, with no need for a skilled technician. Other currently available systems either require expensive equipment, are not easily set up, or do not communicate with a supervising physician. The rehabilitation process of poststroke injuries is physically, financially, and mentally burdensome. Without proper care, patients find it difficult to cope with their injuries. With training, the patient’s family can care for the patient with little added risk, as this would elevate the patient’s mental wellness [49,50,51].

The demonstrated system was not without its limitations. It was only able to assess the performance of a single hand. While it is possible to add further exercises and equipment, the cost will proportionally increase. Additionally, it is not easy for stroke patients with limited hand movement to wear this glove, even with help. The differing hand sizes can also affect the readings; hence, the dataset was taken and tested on people with similar hand sizes. However, these disadvantages are present in almost all data gloves.

The future work for this system will focus on applying more visualization and monitoring of kinematic features by accessing the data stored in the database. Moreover, a more modular glove design will be implemented to assist in data gathering and patient comfort. Finally, more exercises, features, and classifiers will be utilized to cover more FMA exercises or even other assessment methods that can assist in home rehabilitation.

## Figures and Tables

**Table 1:** Sensor data.

Sensor	Readings
Flex	Bending
Force	Gripping Force
IMU	Acceleration and Orientation

**Table 2:** Patient description.

No.	Age/Gender	Description
1	77/M	Uncontrolled diabetes and generalized weakness
2	65/F	Suspected motor neuron syndrome
3	91/M	Parkinson’s

**Table 3:** XGB and LR accuracy for grasping.

Features	XGBoost	LR
All	90.00% ± 9.35%	87.50% ± 7.91%
STD and Min	90.00% ± 9.35%	92.50% ± 10.00%
Mean and RMS	82.50% ± 10.00%	85.00% ± 9.35%
STD and RMS	92.50% ± 6.12%	87.50% ± 7.91%
Mean and Min	85.00% ± 14.58%	90.00% ± 5.00%
**Average**	88.00% ± 9.88%	88.50% ± 8.03%

**Table 4:** XGB and LR accuracy for pinching.

Features	XGBoost	LR
All	82.50% ± 10.00%	90.00% ± 9.35%
STD and Min	90.00% ± 9.35%	85.00% ± 18.37%
Mean and RMS	80.00% ± 10.00%	82.50% ± 6.12%
STD and RMS	90.00% ± 9.35%	87.50% ± 7.91%
Mean and Min	75.00% ± 0.00%	70.00% ± 12.75%
**Average**	83.50% ± 7.74%	83.00% ± 10.90%

**Table 5:** XGB and LR accuracy for waving.

Features	XGBoost	LR
All	90.00% ± 9.35%	90.00% ± 9.35%
STD and SMA	90.00% ± 9.35%	87.50% ± 11.18%
Mean and RMS	77.50% ± 26.69%	87.50% ± 11.18%
STD and RMS	85.00% ± 5.00%	85.50% ± 18.37%
Mean and SMA	67.50% ± 23.18%	82.50% ± 12.75%
**Average**	82.00% ± 14.71%	86.50% ± 12.57%

**Table 6:** XGBoost performance.

	Grasp	Pinch	Wave
BPF	STD and RMS	ALL	ALL
BPF Accuracy	53%	78%	90%
**Average**	33%	78%	80%

**Table 7:** LR performance.

	Grasp	Pinch	Wave
BPF	STD and RMS	Mean and RMS	ALL
BPF Accuracy	87%	78%	70%
**Average**	56%	60%	54%

**Table 8:** Comparison to other systems.

System	Proposed System	Sheng, B. et al. [18]	Song, X. et al. [21]	Otten, P. et al. [22]	Lin, B.S. et al. [28]
**Equipment**	data glove	Kinect V2	Cellphone	Kinect, pressure sensor, Data glove, IMU	Data glove
**Data**	Finger bending angle, gripping force	Upper body	Position, orientation, acceleration	Upper body kinematic data, gripping force	Hand acceleration, angular velocity, angle
**Assessment**	FMA	WMFT-FAS [52]	FMA	FMA	Brunnstrom [53]
**Classification Method**	Machine learning	Machine learning	Decision tree	Machine learning	Machine learning
**IoT Monitoring**	Yes	No	No	No	No
**Setting**	Clinic or in-home	Clinic	Clinic or in-home	Clinic	Clinic
**Accuracy**	85%	92%	85%	61.73%	70.22%

## Data Availability

The data that support the findings of this study are openly available in an online repository called iGrasp_DataGlove at https://github.com/HSarwat/iGrasp_DataGlove (accessed on 16 September 2021).
